# LSD1 as a Biomarker and the Outcome of Its Inhibitors in the Clinical Trial: The Therapy Opportunity in Tumor

**DOI:** 10.1155/2021/5512524

**Published:** 2021-03-25

**Authors:** Clement Agboyibor, Jianshu Dong, Clement Yaw Effah, Emmanuel Kwateng Drokow, Waqar Pervaiz, Hong-Min Liu

**Affiliations:** ^1^School of Pharmaceutical Sciences, Zhengzhou University, Zhengzhou 450001, China; ^2^Co-Innovation Center of Henan Province for New Drug R & D and Preclinical Safety, Zhengzhou 450001, China; ^3^Key Laboratory of Advanced Technology of Drug Preparation Technologies (Zhengzhou University), Ministry of Education of China, Zhengzhou 450001, China; ^4^College of Public Health, Zhengzhou University, Zhengzhou 450001, China; ^5^Department of Oncology, Zhengzhou University People's Hospital and Henan Provincial People's Hospital Henan, Zhengzhou 450003, China

## Abstract

Tumors are the foremost cause of death worldwide. As a result of that, there has been a significant enhancement in the investigation, treatment methods, and good maintenance practices on cancer. However, the sensitivity and specificity of a lot of tumor biomarkers are not adequate. Hence, it is of inordinate significance to ascertain novel biomarkers to forecast the prognosis and therapy targets for tumors. This review characterized LSD1 as a biomarker in different tumors. LSD1 inhibitors in clinical trials were also discussed. The recent pattern advocates that LSD1 is engaged at sauce chromatin zones linking with complexes of multi-protein having an exact DNA-binding transcription factor, establishing LSD1 as a favorable epigenetic target, and also gives a large selection of therapeutic targets to treat different tumors. This review sturdily backing the oncogenic probable of LSD1 in different tumors indicated that LSD1 levels can be used to monitor and identify different tumors and can be a useful biomarker of progression and fair diagnosis in tumor patients. The clinical trials showed that inhibitors of LSD1 have growing evidence of clinical efficacy which is very encouraging and promising. However, for some of the inhibitors such as GSK2879552, though selective, potent, and effective, its disease control was poor as the rate of adverse events (AEs) was high in tumor patients causing clinical trial termination, and continuation could not be supported by the risk-benefit profile. Therefore, we propose that, to attain excellent clinical results of inhibitors of LSD1, much attention is required in designing appropriate dosing regimens, developing in-depth in vitro/in vivo mechanistic works of LSD1 inhibitors, and developing inhibitors of LSD1 that are reversible, safe, potent, and selective which may offer safer profiles.

## 1. Introduction

Epigenetic modification is vital for physiological progress and steady-state gene expression in eukaryotes [1] and is required for numerous biological developments that range from gene expression to disease pathogenesis [2]. DNA methylation, histone modifications, and posttranslational modifications (PTMs) characterize epigenetic variations that may, alone or in combination, modify chromatin structure and gene activity by expediting either gene activation or suppression depending on the regulator type [3]. LSD1 was initially identified by Shi et al. [4] and is a monoamine oxidase homolog that precisely removes H3 from H3K4 and H3K9, thus triggering activation and suppression of genes [5]. Currently, it has been proven that LSD1 demethylates H4K20 and contributes to the balance of numerous additional methylated lysine remains in histone H3 [6].

LSD1 functions as a transcription co-repressor by demethylating H3K4me2/1 and shaping chromatin into a repressive conformation through diverse complexes formed by LSD1 and other numerous proteins. Again, LSD1 functions as a demethylase of non-histone protein by minimizing the reaction of p53 and 53BP1, a tumor suppressor gene, by removing a methyl group from p53K370me2, thereby suppressing the role of p53 [7]. It is well known that LSD1 is upregulated in numerous tumors [8]. Aberrantly, LSD1 upregulation stimulates tumorigenesis by regulating chromatin remodeling and aggregation [9]. Furthermore, the LSD1 upregulation affected the cell cycle of cancer [10] which can result in the inhibition of the p53 tasked to inhibit the reaction between TP53BP1 and p53, thus p53 binding protein 1 [11], which then enhances the growth of cancer, invasion, and metastasis by affecting the methylation/demethylation process [7, 12, 13]. Hence, LSD1 is becoming a significant therapeutic target for cancer treatment [14]. LSD1 upregulation has been observed in various hematological ailments, including AML, ALL, CML, and myelodysplastic syndrome (MDS) [15]. LSD1 upregulation has also been observed in solid tumors, including neuroblastoma, CRC, NSCLC, and breast tumor [16]. These outcomes have sustained LSD1 in its oncogenic activity in both hematological and solid tumors making it a suitable target for cancer treatment. To determine a tumor onset and progression, efficacy of drug treatment, and patients' susceptibility to develop a certain type of tumor, and also predict the efficacy of treatment at a particular tumor stage require a biomarker, therefore, this review characterized LSD1 as a biomarker in tumors. The outcome of LSD1 inhibitors in clinical trials was also discussed.

## 2. LSD1-Protein Interactions in Different Tumors

**Figure 1:**
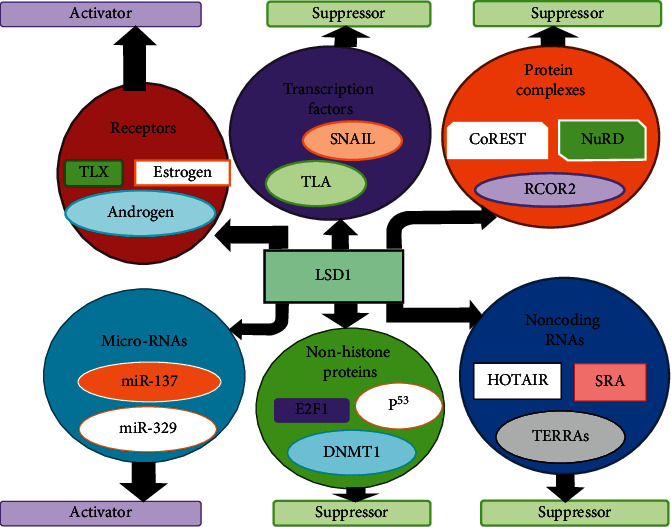
LSD1 interacting partners and functional diversity [18]. LSD1 contains several protein complexes (such as “NuRD and RCOR2”), receptors (such as “estrogen and androgen”), noncoding RNAs (such as “HOTAIR and SRA”), microRNAs (such as “miR-137 and miR-329”), non-histone proteins (such as “p53 and E2F1”), and transcription factors (such as “TLA and SNAIL”). The reaction of LSD1 among varied factors permits the varied directive of diverse biological procedures via the suppression and the activation of target gene expression subject on the mode of its interrelating partners; thus, LSD1 is downregulated when there is the reaction of LSD1 with miR-137 and results in cell differentiation by activating the linked genes. When associated with CoREST, it results in downregulation of LSD1 and suppresses target genes.

Interaction among proteins increases the selection of therapy targets [17]. However, they are difficult to be addressed with conventional small molecule type drugs and often require “beyond rule of 5” type molecules for potent inhibition. The interactions of LSD1 with other proteins in current research are summarized below. Protein complexes such as “NuRD and RCOR2,” non-histone proteins such as “p53 and E2F1,” transcription factors such as “TLA and SNAIL,” receptors such as “estrogen and androgen,” and noncoding RNAs such as “HOTAIR and SRA” are the interacting partners of LSD1 and LSD1 functional diversity rests on them (Figure 1) [18].

p53, which is the controller of numerous cellular life processes such as “programmed cell death, cell cycle progression, and genomic stability” [19], displays various methylated lysine residues where K370 plays a significant role with each level of its methylation showing diverse biological impacts [20]. LSD1 reacts with p53 to suppress p53-mediated transcriptional activation and to inhibit the role of p53 in promoting apoptosis. Also, it was detected that LSD1 removes K370me1 and K370me2 at K370. Huang et al. further indicated that while K370me1 suppresses the p53 role, K370me2 stimulates the link with the co-activator 53BP1 via tandem Tudor domains in 53BP1 [11].

Zinc-finger protein 217 (ZNF217) which function as part of a transcriptional repressor complex regularly increases in cancers; “breast tumor and colorectal cancer” and upregulated ZNF217 is typically linked with poor prognosis [21]. A study by Si et al. [22] stated that ZNF217 links with upregulated LSD1 in HCC cells and directly interacts with and effectively leaps to the whole length of LSD1 in vitro. The study further postulated that ZNF217 could stimulate HCC advancement by employing LSD1 to reduce the H3K4me2 level at the CDH1 promoter and repressing CDH1 transcription.

LSD1 reacts with CtBP [23], which are renowned suppressors of mammalian gene expression [24]. LSD1 is also associated with variability of CtBP roles, including the control of the progress of the pituitary gland [25], suppression of BRCA1 which is considered to be a tumor-suppressor gene [26], and activation of tissue-specific genes in endocrine cells in the gastrointestinal tract [27]. However, the suppression of E-cadherins, proteins involved in the process of EMT, is the more well-known role of the LSD1 and CtBP [23].

Moreover, Lin et al. [28] reported that Snail made use of its extremely well-maintained SNAG domain as a pseudo-substrate to attract LSD1 to its target gene for transcription repression and EMT induction. The study again detected that the expression of Snail meaningfully links with LSD1 expression in multiple human breast tumor tissues. Serrano-Gomez et al. [29] and Ferrari-Amorotti et al. [30] postulated that LSD1 links with SNAIL1 in breast tumors. Li et al. [31] discovered that LSD1 interrelates with the promoter of E-cadherin and demethylated histone H3 lysine 4 (H3K4), downregulated E-cadherin expression, and consequently enhanced ovarian cancer cell migration.

Zang et al. [32] demonstrated that two lncRNAs have been established to have an oncogenic role relating with LSD1: Linc01133, which normalizes the transcription of KFL2, p21 and E-cadherin directing cell proliferation, migration, and invasion as well as apoptosis in NSCLC; and FEZF1-AS that epigenetically suppresses the expression of E-cadherin enhancing EMT procedure [33].

Again, in pancreatic cancer cells (PC) and colorectal cancer tissue (CRC), HOXA cluster antisense RNA2 lncRNA (HOXA-AS2) is complicated in cell growth developing complex with LSD1 [34, 35]. The lncRNA IRAIN forms a complex with LSD1 in PC cells, subduing apoptosis and encouraging proliferation [36]. Three lncRNAs (LINC00673, FOXD2-AS1, HOXA11-AS) have been identified to interact with LSD1 in gastric cancer cells. In particular, the lncRNAs LINC00673 [37] and FOXD2-AS1 [38], in association with LSD1, repress LAST2/KLF2 and EphB3 tumor suppressors, respectively. Furthermore, in cholangiocarcinoma (CCA) and osteosarcoma tumors, the SPRY4-IT1 [38] as well as the FOXP4-AS1 [39] lncRNAs have been shown to assist in tumor growth. Systematically, SPRY4-IT1 employs EZH2 and LSD1 or DNMT1 to KLF2 and LATS2 promoter regions inducing epigenetic silencing [38]. Contrariwise, OS cell growth is sustained when FOXP4-AS1 lncRNA forms complexes with LSD1 and EZH2 and repressing LATS1 transcription [39].

Additionally, a study by Hakimi et al. [40] acknowledged a protein complex connecting LSD1, CoREST (RCOR1), HDAC1, HDAC2, ZNF217, PHF21A, and HMG20B, often termed as the CoREST transcription repressor complex. LSD1 and CoREST are often found in numerous larger protein complexes, within which it acts as a scaffold by linking the deacetylase and demethylase actions into a solitary complex [23]. The link of LSD1 with the CoREST complex permits it to demethylate the nucleosome [41]. In addition to CoREST, CoREST2 and CoREST3 similarly bind to LSD1 and control the functional happenings of this demethylase upon amalgamation into larger protein complexes [42]. However, CoREST2 shows a reduced capacity to enable LSD1-mediated nucleosome demethylation [43]. Unlike CoREST2, competitive inhibition of LSD1-mediated nucleosomal demethylation is detected for CoREST3; as a result, it shows even tougher antagonistic behavior [42]. LSD1 has also been identified in a complex with (“ZMYM2, ZMYM3, GSE1, and GTF2I”) [40], (“CTBP1, HMG20A, HSPA1A, PHF21B, RCOR3, and RREB1”) [18], members of the Mi-2/nucleosome remodeling and deacetylase (NuRD) complex [44], and the lysine methyltransferase mixed-lineage leukemia (MLL) co-activator complex [45]. LSD1 protein complex with CoREST/NuRD is vital for LSD1 to demethylate nucleosomes. LSD1 cannot demethylate nucleosomes alone and thus needs association with RCOR1 or MTA2 in each respective complex [18]. The LSD1 and RCOR1 interaction also protect LSD1 from proteasomal degradation. LSD1 complex with HDAC1/HDAC2 may remove local histone acetylation marks which inhibit the demethylase activity of LSD1 [46].

**Figure 2:**
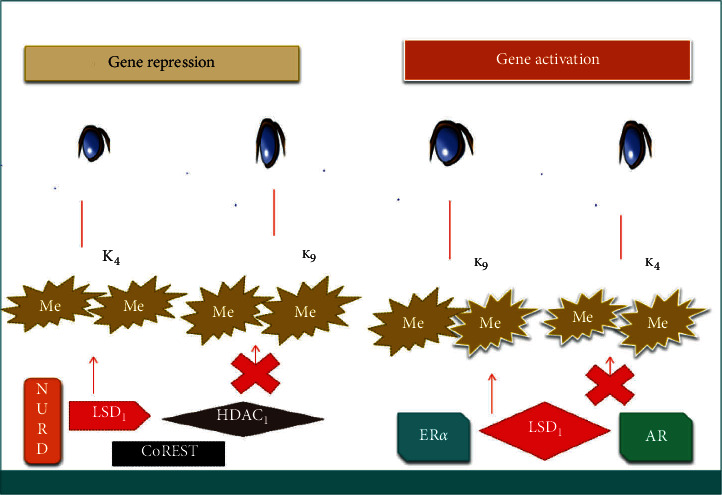
Substrate detailed and directive of gene expression by LSD1. For targeted gene expression inhibition, the removal of H3 from H3K4me1/2 was attained by binding LSD1 to CoREST and NuRD complex. The binding of LSD1 to the CoREST and NuRD complex did not speed up the removal of H3 from H3K9me1/2. For targeted gene expression control, the modification of substrate specificity from H3K4me1/2 to H3K9me1/2 was attained by LSD1 binding to androgen and estrogen receptors.

LSD1 again partakes in nuclear hormonal signaling by reacting with androgen receptors (ARs) [47] as well as estrogen receptors (ERs). ARs are linked with the control of prostate function, from normal tissue advancement to the origination and progression of metastasis [48]. The link of LSD1 with ARs changes its substrate specificity from H3K4me2 to H3K9me1/2 (Figure 2) [47]. LSD1 also interacts with estrogen receptor alpha (ER*α*), which is linked with estrogen signaling in estrogen-responsive tissues, and any deficiency in its role can bring about the initiation and progression of various tumor types [49, 50]. LSD1 functions as both an activator and repressor of genes in association with ER*α*, similar to the mechanism by which LSD1 associates with ARs [51]. From the findings, LSD1 interacts with several proteins within different tumor cells. Therefore, we could confirm that LSD1-protein interaction could provide enormous varieties of therapeutic targets.

## 3. Chemoresistance and LSD1

Chemoresistance is one of the key challenges in tumor treatments and it has largely demonstrated the insensitivity of tumors to therapy, which is an important factor that fails anti-tumor chemotherapy. The available treatment does not give a suitable solution to the resistance of the drug, so more efficient methods are urgently needed to advance the present therapy regimens. The efforts to regain the sensitivity of available chemotherapeutic drugs and subdue resistance of drug of tumor cells are still ongoing [52]. LSD1 is linked with multiple tumor types, and its expression in various tumors is linked with chemoresistance [53–55]. Epithelial-mesenchymal transition (EMT) plays a vital role in the chemoresistance of bladder cancer. The chemoresistance of bladder tumor to therapeutic agents has implicated LSD1. Upregulated LSD1 was detected in chemotherapy bladder tumors and is linked with bladder tumor grades, metastasis status, and prognosis. LSD1 downregulation suppressed not only the EMT process but also cancer development [56]. Lei et al. [55] reported that the upregulation of leucine-rich repeat-containing G-protein-coupled receptor 5 (Lgr5) is linked with the progression of HCC. Lgr5+ HCC cells behave similarly to cancer-initiating cells (CICs) which are highly tumorigenic and resistant to chemotherapeutic agents. Importantly, Lgr5+ cells express higher levels of LSD1, which in turn regulates Lgr5 expression and promotes the self-renewal and drug resistance of Lgr5+ CICs. This finding justifies the design of new therapy approaches, such as drug combination involving LSD1 inhibitors and chemotherapeutic drugs, for the intervention of HCC.

Verigos et al. [53] treated the aggressive types of breast tumor cells, MCF-7 and MDA-MB-468, that have developed therapy resistance with LSD1 inhibitor, GSK-LSD1, and gave them increasing doses of doxorubicin (0–5 *µ*M). Remarkably, the drug's impacts on cell proliferation were substantially enhanced after it was pre-treated with LSD1 inhibitor. Specifically, the IC_50_ values for doxorubicin reduced substantially from 0.64 to 0.28 *µ*M in MCF-7 cells and from 0.37 to 0.26 *µ*M in MDA-MB-468 cells upon pre-treatment with GSK-LSD1. These outcomes propose that LSD1 confers doxorubicin resistance to breast tumor cells and that downregulation of LSD1 makes the cells more sensitive to chemotherapy. The study by Shao et al. supports that LSD1 upregulation enhances proliferation of cells and migration capacity of ovarian tumor cell, SKOV3, and that LSD1 levels might be closely linked to the effects of cisplatin. While upregulated LSD1 confers cisplatin resistance, its downregulation significantly enhances the impacts of cisplatin. Furthermore, cisplatin may directly downregulate LSD1 protein expression in a dose-response manner, suggesting that LSD1 is a downstream target of cisplatin. Thus, cisplatin may inhibit cell proliferation by modulating the epigenetic factor, LSD1 [57].

## 4. Is LSD1 a Tumor Biomarker in Different Tumors?

Some individuals' DNA has detectable genes that can show a higher risk of developing certain tumors. For instance, an individual is at a higher risk of getting “breast tumor,” “ovarian tumor,” and “prostate tumor,” when inheriting the supposed “breast tumor genes,” certain mutations in BRCA1 and BRCA2. Again, children are at high risk of getting ependymoma via a rare inherited condition, neurofibromatosis type 2 (NF2). However, almost all tumors are not inherited and the majority of the people who are diagnosed with tumors do not have any of the supposed “tumor genes.” But all tumors do have biomarkers, including genetic biomarkers.

Carcinogenesis is multiple procedures that involve genetic and epigenetic variations. Epigenetic variations such as histone modifications are possibly reversible procedures; to detect new therapies and diagnostic and prognostic tools in tumors, the mechanism under the epigenetic variations has received a lot of attention towards its understanding. The epigenetic variations when well understood will lead to new tumor-associated gene identification that may characterize attractive targets for tumor treatment and provide new instincts into the biology of tumors [58]. LSD1 plays a crucial role in the regulation of gene expression by removing the methyl groups from methylated lysine 4 of histone H3 and lysine 9 of histone H3. LSD1 partakes in numerous biological processes and is important in the development of mammals. Taking into consideration the current indication that LSD1 is implicated in carcinogenesis, several research works have been done investigating the role of LSD1 in different tumors.

For solid tumors, LSD1 is implicated in various types of solid tumors and its enhanced expression is linked with a poor prognosis. Upregulated LSD1 is associated with undifferentiated and aggressive neuroblastoma and is linked with poor prognosis [13]. LSD1 expression in lung cancer cells is advanced than in usual lung tissue and overexpressed LSD1 is linked with poor prognosis in NSCLC and promoted tumor cell proliferation, migration, and invasion [9]. In NSCLC, the LSD1 inhibitor, pargyline, that downregulates LSD1 expression, determines the suppression of cell growth, migration, and invasion [59]. LSD1 was investigated in breast tumors and detected upregulated LSD1 in estrogen receptor- (ER-) negative tumors [60] and in basal-like breast tumors [61] and it was stated that LSD1 is a prognostic factor of poor outcome in these subtypes. Wu et al. [62] postulated an upregulated LSD1 in hepatocellular carcinoma (HCC) in liver tissues. The upregulated LSD1 is linked with higher cancer stage and higher cancer grade as well as reduced survival time in HCC patients. LSD1 immunoreactivity is enhanced in a substantial fraction of hepatocarcinoma and LSD1 downregulation in hepatocarcinoma cells reduces cell proliferation substantially [59]. Again, the study by Wu et al. [63] stated that LSD1 upregulation in “colon cancer,” “hepatocellular carcinoma,” “human melanomas,” and “tongue cancer” is poorly linked with overall survival. Cancer stem cells (CSCs) within each tumor can initiate tumor growth [64, 65] and are presented with self-renewal, proliferation being infinite, and a possibility of multiple directional differentiation characteristics [66]. LSD1 is implicated in CSCs in most solid tumors as it maintains cancer stemness in tumors including glioblastoma, breast tumor, and HCC [67]. Some studies stated that LSD1 sustains tumor stemness via upregulating the markers of stemness such as “SOX2 and OCT4” in colorectal cancer [68] thus making LSD1 a therapeutic target.

For acute myeloid leukemia, it has been the focus of substantial research in current years. The clonal ailments of hematopoiesis in which LSCs cultivated limitless self-renewal ability, improved proliferation, and impaired hematopoietic differentiation programs are known as acute leukemia. According to FAB classification when comparing LSD1 level in less differentiated subtypes of AML with other subtypes characterized by a higher degree of morphological differentiation, less differentiated subtypes of AML such as the M1 subtype expressed upregulated LSD1 [69]. Harris et al. [70] and Somervaille and Cleary [71] postulated that LSD1 is vital for the progress and maintenance of AML, and in particular of the leukemia stem cell (LSC) compartment, in a mouse model of leukemia caused by the fusion protein MLL-AF9. The significance of LSD1 as a controller of probable LSC has again been explained in models of a mouse and human MLL-AF9 Leukemia [72]. The degree of LSD1 inhibition was meaningfully linked with loss of the LSC probable of AML cells via deterioration of differentiation and apoptosis. Cells with inactive LSD1 are unable to form colonies and show differentiated cell morphology and are not able to cause leukemia when introduced into mice [70]. However, LSD1 has also been shown to be understated in more differentiated AML subtypes such as the M3 FAB subtype and acute promyelocytic leukemia; still, experiments using small molecules propose that LSD1 still has a vital role in controlling AML cell differentiation even in cells that are not strictly reliant on LSD1 for survival. Again, other researchers show depletion and inhibition of LSD1 impairs proliferation in myelodysplastic syndrome (MDSs), acute erythro-leukemia, and acute megakaryoblastic leukemia by induction of cell differentiation [73, 74]. The applicable role of upregulated LSD1 in AML has been validated by the linked outcomes attained in a cytogenetically distinct subtype of AML, the APL, described by a translocation containing promyelocytic leukemia gene, PML and the retinoic acid receptor, RAR*α* genes. All-trans-retinoic-acid (ATRA) as a therapy for this leukemia promotes differentiation of leukemic cells; some APL subtypes are resistant to ATRA. However, other inhibitors of LSD1 including TCP induce morphological and immunophenotypic differentiation of APML cells in vitro. The discoveries that LSD1 plays a key role in AML development drive the efforts to appreciate the genetic program controlled by LSD1 [75]. These outcomes sturdily back the oncogenic probable of LSD1 in AMLs and in particular its capability to sustain LSCs, making it an attractive target for tumor therapy. However, the mechanisms underlying the role of LSD1 at its target genes and the protein complexes employed by LSD1 need additional investigations. Currently, the non-enzymatic role of LSD1 in AML has been revealed by Vinyard et al. [76] via CRISPR-suppressor scanning and it was clarified that AML survival was not dependent on the enzymatic activity of LSD1. The study by Maiques-Diaz et al. [77] and Vinyard et al. [76] which is in agreement with [78] stated that AML survival was dependent on LSD1 interaction with GFI1 and GFI1b (transcriptional repressors) and that the enzymatic action of LSD1 was not beneficial to the AML cells.

For lymphoid leukemias, Yatim et al. [79] and Lobry et al. [80] stated that T-cell acute lymphoblastic leukemia (T-ALL) caused by the malignant change of T-cell progenitors, mutations in Notch1, foremost to aberrant and constitutively vigorous Notch1 signaling, add to oncogenic transformation and are hallmarks of this ailment. T-ALL accounts for about 15 and 25% of ALL in pediatric and adult associates, respectively. Mutations in notch1 are regularly found in T-ALL, resulting in the constitutive activation of the Notch pathway. LSD1 has two roles, functioning as an activator and repressor in Notch-mediated T-ALLs. LSD1 plays a role as a co-repressor when linked with the CSL-repressor complex by removing the H3K4me2 marks at Notch targets in the absence of Notch. However, LSD1 acts as a NOTCH1 co-activator upon Notch activation by ensuring efficient H3K9me2 demethylation [79]. According to Yatim et al. [79], CSL binds and suppresses Notch targets in the absence of Notch, though the existence of Notch converts CSL in a transcriptional activator. The discovery that LSD1 relates with CSL elucidates the mechanism via which Notch controls gene repression by deleting the H3K4me2 marks at Notch targets in the absence of Notch. Certainly, a useful shift of LSD1 action is perceived upon activation of Notch. In the absence of Notch, H3K4me2 demethylation is activated by LSD1 while in its presence the enzyme acts favorably on H3K9me2 resulting in making the target genes active and effective. So, LSD1 inhibition in T-ALL replicates cell growth seizure and alteration of growth, and a phenotype was earlier accredited to Notch silencing. Li et al. [81] and Su et al. [82] postulated that upregulated TAL1 is observed in about 40% of T-ALL. TAL1 requires the LSD1-CoREST complex to repress its target genes in T-ALL. Huang's test center has proven that LSD1 is linked with TAL1/SCL whose dysregulation has been linked with T-cell leukemogenesis. LSD1/Tal1 relation is interrupted by phosphorylation of serine 172 in TAL1 by protein-kinase-A (PKA) and the disrupted TAL1-LSD1 interaction leads to promoter H3K4 hypermethylation and activation of target genes [80]. Therefore, PKA-dependent dynamical action involving LSD1 and TAL1 has a fundamental role in hematopoiesis and leukemogenesis. However, LSD1 may acquire oncogenic roles through numerous mechanisms in T-cell leukemias.

LSD1 upregulation is expressed in most tumor cells and its inhibition prevented tumor cell growth and migration. The upregulated LSD1 is linked with higher cancer stage and higher cancer grade as well as reduced survival time in tumor patients. Therefore, LSD1 levels could be used to monitor and identify different tumors as well as improvement in tumor treatment. So, we could predict that LSD1 is a biomarker for most tumors.

## 5. The Outcome of Some LSD1 Inhibitors in Clinical Trials

The role of LSD1 during carcinogenesis is highly significant and targeting LSD1 has become an emerging option for tumor treatment. So, researchers in the past decade have come up with several pharmacological inhibitors of LSD1 that are potent, effective, and selective. These inhibitors can be grouped as natural and synthetic and subcategorized into irreversible and reversible [83, 84]. A lot of the irreversible LSD1 inhibitors (TCP, ORY-1001 [85], GSK-2879552 [86], IMG-7289, INCB059872 [87], and ORY-2001) are presently undergoing clinical trials. CC-90011, which is a reversible LSD1 inhibitor, is also being assessed in clinical trials (Table 1). Here, we review the outcome of available LSD1 inhibitors that have undergone clinical trials.

### 5.1. GSK2879552

GSK2879552 as an LSD1 inhibitor for tumor (relapsed/refractory (R/R) AML (NCT02177812) and SCLC (NCT02034123) malignancies) treatments was in phase I clinical studies. For the outcome of the single-agent GSK2879552 in relapsed/refractory SCLC malignancy, twenty-nine patients were apportioned for this trial. 22 patients were able to complete the study; due to adverse events (AEs), 7 patients withdrew from the study. 83% of the patients had at least one treatment-associated AE. Thrombocytopenia was the most common treatment-associated AE and 41% of the patients experienced it. Nine patients gave an account of 12 serious AEs (SAEs), and 6 were treatment-related with encephalopathy (four SAEs) being the most common. The study recorded 3 deaths and one was linked to serious AEs. PK was characterized by quick absorption, slow deletion, and a dose-proportional rise in exposure [88]. For relapsed/refractory AML malignancy, double-agent, GSK287955, and All-Trans Retinoic Acid (ATRA) were used to evaluate recommended phase II dose (RP2D) and regimen for the orally administered GSK2879552, alone or in combination with ATRA. The trial was in two stages. Stage 1 considered maximum tolerated dose (MTD) and/or RP2D making use of the dose-escalation procedure. Stage 2 is to explore the safety, tolerability, and clinical activity of GSK2879552, alone or in combination with ATRA, at the RP2D in subjects with AML. However, the phase 2 study did not occur since the phase I study was terminated at the early stages [89].

### 5.2. CC-90011

CC-90011 inhibitor was reported to be in phase I trial, evaluated in solid tumors and R/R non-Hodgkin's lymphomas (NCT02875223) [90]. 50 patients were used in the study; 49 had solid tumors, 1 had R/R non-Hodgkin's lymphomas, and 26 had neuroendocrine neoplasms (NENs). Patients were treated with escalating doses of CC-90011 at 1.25, 2.5, 5, 10, 20, 40, 60, 80, and 120 mg with their corresponding number of patients (*n* = 4), (*n* = 5), (*n* = 6), (*n* = 4), (*n* = 5), (*n* = 6), (*n* = 6), (*n* = 10), and (*n* = 4). 16% and 8% of the patients had thrombocytopenia and neutropenia, respectively, being the most common treatment-associated AEs. 8% of the patients having thrombocytopenia were as a result of doses being too high. 40% of the patients experienced serious AEs and six percent were treatment-associated. Two to four hours after dose, the peak plasma concentrations occurred and the mean terminal half-life was 60 hours; the exposure was dose-proportional. PD analysis showed decreased CgA and MMD in response to CC-90011, correlating with clinical benefit. One patient achieved a complete response (CR) and 22 had stable disease (SD). Prolonged SD 4 months occurred in 7 patients, 5 with bronchial NEN and 2 with prostate NEN [90].

### 5.3. IMG-7289

Phase IIb trial evaluated single-agent IMG-7289 in essential thrombocythemia (NCT04081220) [91] and myelofibrosis (NCT03136185) [92]. Phase I/IIa trial evaluated single-agent/double-agent IMG-7289 in AML and myelodysplastic syndrome (NCT03136185) [93]. For AML and myelodysplastic syndrome, the phase I manifold rising dose quota of the trial assessing IMG-7289 as a single-agent was magnificently completed. The study advanced into the phase IIa development arm which assessed IMG-7289/ATRA combo treatment regimen for prolonged dosing periods. Treatment of the final IIa expansion cohort is still ongoing [93]. In the case of myelofibrosis, the data establishes the prospects of IMG-7289 as a single-agent in intermediate-2 and high-risk patients of myelofibrosis that are intolerant to Janus Kinase (JAK) inhibitor. IMG-7289 is now at phase II trials. Clinical endpoints include “spleen volume reduction, reduction in total symptom scores, and improvement in circulating inflammatory cytokines, anemia and bone marrow fibrosis and blast count” [92]. As in essential thrombocythemia, the phase II study deals with how effective IMG-7289 functions in the treatment of essential thrombocythemia. IMG-7289 plays an important role by ceasing LSD1 action. Upregulated LSD1 in essential thrombocythemia patients is known for the development of abnormal cells. IMG-7289 lowers the abnormal red cell and platelet counts seen in patients with essential thrombocythemia. IMG-7289 may decrease spleen size and other inflammatory markers which are believed to cause symptoms in these diseases [92].

### 5.4. Tranylcypromine (TCP)

Phase I/II trial evaluated double-agent TCP in refractory/relapsed AML (NCT02261779) [94] and triple-agent TCP in non-APL AML/MDS (NCT02717884) [95]. The clinical study of TCP/ATRA treatment phase I/II explored safety and efficacy, for R/R AML. The combo trial was estimated in eighteen patients that do not meet the requirements for intensive treatment. Twenty percent total retort rate with two complete remissions devoid of hematological recovery and one partial retort were observed. The TCP/ATRA combo treatment showed myeloid differentiation in patients without clinical remission. The median OS was 3.3 months, and 1-year OS was 22%. Differentiation syndrome which is ATRA-induced was developed by one patient. Vertigo and hypotension were considered the utmost recurrently AE. There is a correlation between TCP plasma levels and intracellular TCP concentration. In the AML blasts and white blood cells of some of the patients treated with TCP/ATRA combo were observed to have upregulated H3K4me1 and H3k4me2. Differentiation of AML blasts could be induced by drug combination treatment of TCP/ATRA and result in clinical response in heavily pre-treated patients with refractory/relapsed AML with acceptable toxicity [94]. For non-APL AML/MDS, the clinical study phase I is the assessment of MTD of tranylcypromine (TCP) together with fixed-dose ATRA and Cytarabine (AraC) to derive the recommended phase II dose (RP2D) in patients with non-APL AML/MDS for whom no standard treatment is available. The drug combination of TCP with fixed-dose ATRA and AraC in phase II clinical study is to assess the efficacy of TCP at the RP2D. It is the first efficacy evaluation to pave way for further investigations of TCP [95]. The phase I clinical trial of TCP and ATRA on non-APL and AML was carried out based on a paper published in Nature Medicine with their hypothesis supported by the preliminary data that non-APL and AML cells can be re-sensitized to ATRA when combined with LSD 1 agents.

### 5.5. ORY-1001

Clinical study phase II estimated double-agent ORY-1001 in elderly AML patients (ISIN Code: ES0167733015, ORY) [96] and SCLC patients [97] and phase I/IIa study evaluated single-agent ORY-1001 in R/R acute leukemia patients (EudraCT No.: 2018-000482-36) [78]. Maes et al. [78] stated the early clinical study phase I/IIa with ORY-1001 in R/R acute leukemia patients showed safety and admirable tolerability of the drug and initial signs of anti-leukemic action. The drug combination treatment of ORY-1001 and azacitidine in the phase II study of ORY-1001 with AML patients (elderly) is ongoing with encouraging evidence of clinical efficacy. Eight patients are in this evaluation, out of which 6 patients are attaining objective responses (OR): complete remissions with incomplete hematologic recovery (CRi) in 3 patients, complete remissions in 2 patients, and 1 partial remission patient. Twenty weeks was the average monitored period amongst the evaluable patients with an average time to response of 32 days in those patients who responded. Transfusion independent has happened in 2 out of 5 patients that have been administered with more than 3 cycles of the treatment. The outcome gives support for a substantial synergistic effect from ORY-1001 looking into the 27% historical response rates in this population when treated with azacitidine alone [96]. The Phase IIa clinical combo trial with ORY-1001 has begun on the premises of the preclinical studies; the drug combination of ORY-1001 and platinum etoposide has shown promising outcome. The study is to evaluate the safety, tolerability, dose-finding, and efficacy of ORY-1001 in combo with platinum-etoposide in patients with SCLC [97].

## 6. Conclusion

It has been recognized that LSD1 is existing in numerous transcription factor complexes affecting numerous biological roles depending upon the exact complex in which LSD1 is present. The recent pattern advocates that LSD1 is employed at sauce chromatin zones, interacting with multiple protein complexes having a definite DNA-binding transcription factor, recognizing LSD1 as a favorable epigenetic target, and also gives a large selection of therapeutic targets to treat different tumors. This review sturdily backs the oncogenic probable of LSD1 in different tumors, indicating that LSD1 levels can be used to monitor and identify different tumors and can be a useful biomarker of progression and fair diagnosis in different tumor patients. The clinical trials showed that both the single-agent and the double-agent inhibitors of LSD1 have growing evidence of clinical efficacy which is very encouraging and promising. However, for some of the inhibitors such as GSK2879552, though selective, potent, and effective, its disease control was poor with the rate of adverse events (AEs) being high in tumor patients causing clinical trial termination, as continuation could not be supported by the risk-benefit profile. Therefore, we propose that, to attain excellent clinical results of inhibitors of LSD1, much attention is required in designing appropriate dosing regimens, developing in-depth in vitro/in vivo mechanistic studies of inhibitors of LSD1, and developing inhibitors of LSD1 that are reversible, safe, potent, and selective which may offer safer profiles.

## Figures and Tables

**Table 1:** LSD1 inhibitors in clinical trials.

Inhibitors	Malignancy	Study phase	Study description	Trial number
GSK2879552	Relapsed/refractory SCLC	Phase I	Single-agent	NCT02034123
GSK2879552	Relapsed/refractory AML	Phase I	Double-agent	NCT02177812
CC-90011	Relapsed/refractory solid tumors and non-Hodgkin's lymphomas	Phase I	Single-agent	NCT02875223
IMG-7289	Acute myeloid leukemia and myelodysplastic syndrome	Phase I/IIa	Single-agent/double-agent	NCT03136185
IMG-7289	Essential thrombocythemia	Phase IIb	Single-agent	NCT04081220
IMG-7289	Myelofibrosis	Phase IIb	Single-agent	NCT03136185
TCP	Refractory/relapsed AML	Phase I/II	Double-agent	NCT02261779
TCP	Non-APL AML/MDS	Phase I/II	Triple-agent	NCT02717884
ORY-1001	Elderly AML	Phase II	Double-agent	ISIN code: ES0167733015, ORY
ORY-1001	SCLC	Phase II	Double-agent	N/A
ORY-1001	Refractory/relapsed acute leukemia patients	Phase I/IIa	Single-agent	EudraCT no.: 2018-000482-36

## Abbreviations

AEs: Adverse events
TCP: Tranylcypromine
MTD: Maximum tolerated dose
CRi: Incomplete hematologic recovery
EMT: Epithelial-mesenchymal transition
LSD1: Lysine-specific demethylase 1
PK: Pharmacokinetics
PD: Pharmacodynamics
CICs: Cancer-initiating cells
AML: Acute myeloid leukemia
ALL: Acute lymphoblastic leukemia
CML: Chronic myelogenous leukemia
MDS: Myelodysplastic syndrome
CtBP: C-terminal binding proteins
CRC: Colorectal cancer
CSCs: Cancer stem cells
LSCs: Leukemic stem cells
APL: Acute promyelocytic leukemia.

## References

[1] Xie P., Zang L.-Q., Li X.-K., Shu Q. (2016). An epigenetic view of developmental diseases: new targets, new therapies. *World Journal of Pediatrics*.

[2] Perri F., Longo F., Giuliano M. (2017). Epigenetic control of gene expression: potential implications for cancer treatment. *Critical Reviews in Oncology/Hematology*.

[3] Mohammed S. I., Springfield S., Das R. (2012). Role of epigenetics in cancer health disparities. *Methods in Molecular Biology*.

[4] Shi Y., Lan F., Matson C. (2004). Histone demethylation mediated by the nuclear amine oxidase homolog LSD1. *Cell*.

[5] Forneris F., Binda C., Vanoni M. A., Mattevi A., Battaglioli E. (2005). Histone demethylation catalysed by LSD1 is a flavin-dependent oxidative process. *FEBS Letters*.

[6] Perillo B., Tramontano A., Pezone A., Migliaccio A. (2020). LSD1: more than demethylation of histone lysine residues. *Experimental & Molecular Medicine*.

[7] Sorna V., Theisen E. R., Stephens B. (2013). High-throughput virtual screening identifies NovelN′-(1-phenylethylidene)-benzohydrazides as potent, specific, and reversible LSD1 inhibitors. *Journal of Medicinal Chemistry*.

[8] Maiques-Diaz A., Somervaille T. C. (2016). LSD1: biologic roles and therapeutic targeting. *Epigenomics*.

[9] Hayami S., Kelly J. D., Cho H.-S. (2011). Overexpression of LSD1 contributes to human carcinogenesis through chromatin regulation in various cancers. *International Journal of Cancer*.

[10] Cho H.-S., Suzuki T., Dohmae N. (2011). Demethylation of RB regulator MYPT1 by histone demethylase LSD1 promotes cell cycle progression in cancer cells. *Cancer Research*.

[11] Huang J., Sengupta R., Espejo A. B. (2007). p53 is regulated by the lysine demethylase LSD1. *Nature*.

[12] Yu Y., Wang B., Zhang K. (2013). High expression of lysine-specific demethylase 1 correlates with poor prognosis of patients with esophageal squamous cell carcinoma. *Biochemical and Biophysical Research Communications*.

[13] Lv T., Yuan D., Miao X. (2012). Over-expression of LSD1 promotes proliferation, migration and invasion in non-small cell lung cancer. *PLoS One*.

[14] Lynch J. T., Harris W. J., Somervaille T. C. P. (2012). LSD1 inhibition: a therapeutic strategy in cancer?. *Expert Opinion on Therapeutic Targets*.

[15] Castelli G., Pelosi E., Testa U. (2018). Targeting histone methyltransferase and demethylase in acute myeloid leukemia therapy. *OncoTargets and Therapy*.

[16] Rao M., Chinnasamy N., Hong J. A. (2011). Inhibition of histone lysine methylation enhances cancer-testis antigen expression in lung cancer cells: implications for adoptive immunotherapy of cancer. *Cancer Research*.

[17] Pelay-Gimeno M., Glas A., Koch O., Grossmann T. N. (2015). Structure-based design of inhibitors of protein-protein interactions: mimicking peptide binding epitopes. *Angewandte Chemie International Edition*.

[18] Shi Y.-J., Matson C., Lan F., Iwase S., Baba T., Shi Y. (2005). Regulation of LSD1 histone demethylase activity by its associated factors. *Molecular Cell*.

[19] Li T., Kon N., Jiang L. (2012). Tumor suppression in the absence of p53-mediated cell-cycle arrest, apoptosis, and senescence. *Cell*.

[20] Huang J., Dorsey J., Chuikov S. (2010). G9a and Glp methylate lysine 373 in the tumor suppressor p53. *Journal of Biological Chemistry*.

[21] Rahman M. T., Nakayama K., Rahman M. (2012). Prognostic and therapeutic impact of the chromosome 20q13.2 ZNF217 locus amplification in ovarian clear cell carcinoma. *Cancer*.

[22] Si W., Zhao Y., Zhou J., Zhang Q., Zhang Y. (2019). The coordination between ZNF217 and LSD1 contributes to hepatocellular carcinoma progress and is negatively regulated by miR-101. *Experimental Cell Research*.

[23] Shi Y., Sawada J.-i., Sui G. (2003). Coordinated histone modifications mediated by a CtBP co-repressor complex. *Nature*.

[24] Chinnadurai G. (2007). Transcriptional regulation by C-terminal binding proteins. *The International Journal of Biochemistry & Cell Biology*.

[25] Wang J., Scully K., Zhu X. (2007). Opposing LSD1 complexes function in developmental gene activation and repression programmes. *Nature*.

[26] Banck M. S., Li S., Nishio H., Wang C., Beutler A. S., Walsh M. J. (2009). The ZNF217 oncogene is a candidate organizer of repressive histone modifiers. *Epigenetics*.

[27] Chinnadurai G. (2009). The transcriptional corepressor CtBP: a foe of multiple tumor suppressors: figure 1. *Cancer Research*.

[28] Lin Y., Wu Y., Li J. (2010). The SNAG domain of Snail1 functions as a molecular hook for recruiting lysine-specific demethylase 1. *The EMBO Journal*.

[29] Serrano-Gomez S. J., Maziveyi M., Alahari S. K. (2016). Regulation of epithelial-mesenchymal transition through epigenetic and post-translational modifications. *Molecular Cancer*.

[30] Ferrari-Amorotti G., Chiodoni C., Shen F. (2014). Suppression of invasion and metastasis of triple-negative breast cancer lines by pharmacological or genetic inhibition of slug activity. *Neoplasia*.

[31] Li Y., Wan X., Wei Y. (2016). LSD1-mediated epigenetic modification contributes to ovarian cancer cell migration and invasion. *Oncology Reports*.

[32] Zang C., Nie F.-q., Wang Q. (2016). Long non-coding RNA LINC01133 represses KLF2, P21 and E-cadherin transcription through binding with EZH2, LSD1 in non small cell lung cancer. *Oncotarget*.

[33] He R., Zhang F. h., Shen N. (2017). LncRNA FEZF1-AS1 enhances epithelial-mesenchymal transition (EMT) through suppressing E-cadherin and regulating WNT pathway in non-small cell lung cancer (NSCLC). *Biomedicine & Pharmacotherapy*.

[34] Ding J., Xie M., Lian Y. (2017). Long noncoding RNA HOXA-AS2 represses P21 and KLF2 expression transcription by binding with EZH2, LSD1 in colorectal cancer. *Oncogenesis*.

[35] Lian Y., Li Z., Fan Y. (2017). The lncRNA-HOXA-AS2/EZH2/LSD1 oncogene complex promotes cell proliferation in pancreatic cancer. *American Journal of Translational Research*.

[36] Lian Y., Wang J., Feng J. (2016). Long non-coding RNA IRAIN suppresses apoptosis and promotes proliferation by binding to LSD1 and EZH2 in pancreatic cancer. *Tumor Biology*.

[37] Huang M., Hou J., Wang Y. (2017). Long noncoding RNA LINC00673 is activated by SP1 and exerts oncogenic properties by interacting with LSD1 and EZH2 in gastric cancer. *Molecular Therapy*.

[38] Xu Y., Yao Y., Jiang X. (2018). SP1-induced upregulation of lncRNA SPRY4-IT1 exerts oncogenic properties by scaffolding EZH2/LSD1/DNMT1 and sponging miR-101-3p in cholangiocarcinoma. *Journal of Experimental & Clinical Cancer Research*.

[39] Yang L., Ge D., Chen X. (2018). FOXP4-AS1 participates in the development and progression of osteosarcoma by downregulating LATS1 via binding to LSD1 and EZH2. *Biochemical and Biophysical Research Communications*.

[40] Hakimi M.-A., Dong Y., Lane W. S., Speicher D. W., Shiekhattar R. (2003). A candidate X-linked mental retardation gene is a component of a new family of histone deacetylase-containing complexes. *Journal of Biological Chemistry*.

[41] Lee M. G., Wynder C., Cooch N., Shiekhattar R. (2005). An essential role for CoREST in nucleosomal histone 3 lysine 4 demethylation. *Nature*.

[42] Upadhyay G., Chowdhury A. H., Vaidyanathan B., Kim D., Saleque S. (2014). Antagonistic actions of Rcor proteins regulate LSD1 activity and cellular differentiation. *Proceedings of the National Academy of Sciences*.

[43] Barrios A. P., Gómez A. V., Sáez J. E. (2014). Differential properties of transcriptional complexes formed by the CoREST family. *Molecular and Cellular Biology*.

[44] Wang J., Hevi S., Kurash J. K. (2009). The lysine demethylase LSD1 (KDM1) is required for maintenance of global DNA methylation. *Nature Genetics*.

[45] Nakamura T., Mori T., Tada S. (2002). ALL-1 is a histone methyltransferase that assembles a supercomplex of proteins involved in transcriptional regulation. *Molecular Cell*.

[46] Lee M. G., Wynder C., Bochar D. A., Hakimi M.-A., Cooch N., Shiekhattar R. (2006). Functional interplay between histone demethylase and deacetylase enzymes. *Molecular and Cellular Biology*.

[47] Metzger E., Wissmann M., Yin N. (2005). LSD1 demethylates repressive histone marks to promote androgen-receptor-dependent transcription. *Nature*.

[48] Zhou Y., Bolton E. C., Jones J. O. (2015). Androgens and androgen receptor signaling in prostate tumorigenesis. *Journal of Molecular Endocrinology*.

[49] Thomas C., Gustafsson J.-Å. (2011). The different roles of ER subtypes in cancer biology and therapy. *Nature Reviews Cancer*.

[50] Perillo B., Ombra M. N., Bertoni A. (2008). DNA oxidation as triggered by H3K9me2 demethylation drives estrogen-induced gene expression. *Science*.

[51] Garcia-Bassets I., Kwon Y.-S., Telese F. (2007). Histone methylation-dependent mechanisms impose ligand dependency for gene activation by nuclear receptors. *Cell*.

[52] Ji X., Lu Y., Tian H., Meng X., Wei M., Cho W. C. (2019). Chemoresistance mechanisms of breast cancer and their countermeasures. *Biomedicine & Pharmacotherapy*.

[53] Verigos J., Karakaidos P., Kordias D. (2019). The histone demethylase LSD1/ΚDM1A mediates chemoresistance in breast cancer via regulation of a stem cell program. *Cancers*.

[54] Hollebecque A., Salvagni S., Plummer R. (2020). Phase I study of lysine-specific demethylase 1 inhibitor, CC-90011, in patients with advanced solid tumors and relapsed/refractory non-hodgkin lymphoma. *Clinical Cancer Research*.

[55] Lei Z.-J., Wang J., Xiao H.-L. (2015). Lysine-specific demethylase 1 promotes the stemness and chemoresistance of Lgr5+ liver cancer initiating cells by suppressing negative regulators of *β*-catenin signaling. *Oncogene*.

[56] Xie Q., Tang T., Pang J. (2020). LSD1 promotes bladder cancer progression by upregulating LEF1 and enhancing EMT. *Frontiers in Oncology*.

[57] Shao G., Wan X., Lai W. (2018). Inhibition of lysine-specific demethylase 1 prevents proliferation and mediates cisplatin sensitivity in ovarian cancer cells. *Oncology Letters*.

[58] Risch A., Plass C. (2008). Lung cancer epigenetics and genetics. *International Journal of Cancer*.

[59] Zhao Z.-K., Dong P., Gu J. (2013). Overexpression of LSD1 in hepatocellular carcinoma: a latent target for the diagnosis and therapy of hepatoma. *Tumor Biology*.

[60] Lim S., Janzer A., Becker A. (2010). Lysine-specific demethylase 1 (LSD1) is highly expressed in ER-negative breast cancers and a biomarker predicting aggressive biology. *Carcinogenesis*.

[61] Nagasawa S., Sedukhina A. S., Nakagawa Y. (2015). LSD1 overexpression is associated with poor prognosis in basal-like breast cancer, and sensitivity to PARP inhibition. *PLoS One*.

[62] Wu L.-w., Zhou D.-m., Zhang Z.-y. (2019). Suppression of LSD1 enhances the cytotoxic and apoptotic effects of regorafenib in hepatocellular carcinoma cells. *Biochemical and Biophysical Research Communications*.

[63] Wu J., Hu L., Du Y., Kong F., Pan Y. (2015). Prognostic role of LSD1 in various cancers: evidence from a meta-analysis. *OncoTargets and Therapy*.

[64] Phi L. T. H., Sari I. N., Yang Y.-G. (2018). Cancer stem cells (CSCs) in drug resistance and their therapeutic implications in cancer treatment. *Stem Cells International*.

[65] Elsaba T. M. A., Martinez-Pomares L., Robins A. R. (2010). The stem cell marker CD133 associates with enhanced colony formation and cell motility in colorectal cancer. *PLoS One*.

[66] Zhou Y., Xia L., Wang H. (2017). Cancer stem cells in progression of colorectal cancer. *Oncotarget*.

[67] Karakaidos P., Verigos J., Magklara A. (2019). LSD1/KDM1A, a gate-keeper of cancer stemness and a promising therapeutic target. *Cancers*.

[68] Chen J., Ding J. Z. J., Wang Z., Du J., Wu C. (2020). Knocking down LSD1 inhibits the stemness features of colorectal cancer stem cells. *Brazilian Journal of Medical and Biological Research*.

[69] Goardon N., Marchi E., Atzberger A. (2011). Coexistence of LMPP-like and GMP-like leukemia stem cells in acute myeloid leukemia. *Cancer Cell*.

[70] Harris W. J., Huang X., Lynch J. T. (2012). The histone demethylase KDM1A sustains the oncogenic potential of MLL-AF9 leukemia stem cells. *Cancer Cell*.

[71] Somervaille T. C. P., Cleary M. L. (2006). Identification and characterization of leukemia stem cells in murine MLL-AF9 acute myeloid leukemia. *Cancer Cell*.

[72] Lokken A. A., Zeleznik-Le N. J. (2012). Breaking the LSD1/KDM1A addiction: therapeutic targeting of the epigenetic modifier in AML. *Cancer Cell*.

[73] Sugino N., Kawahara M., Tatsumi G. (2017). A novel LSD1 inhibitor NCD38 ameliorates MDS-related leukemia with complex karyotype by attenuating leukemia programs via activating super-enhancers. *Leukemia*.

[74] Ishikawa Y., Gamo K., Yabuki M. (2017). A novel LSD1 inhibitor T-3775440 disrupts GFI1B-containing complex leading to transdifferentiation and impaired growth of AML cells. *Molecular Cancer Therapeutics*.

[75] Schenk T., Chen W. C., Göllner S. (2012). Inhibition of the LSD1 (KDM1A) demethylase reactivates the all-trans-retinoic acid differentiation pathway in acute myeloid leukemia. *Nature Medicine*.

[76] Vinyard M. E., Su C., Siegenfeld A. P. (2019). CRISPR-suppressor scanning reveals a nonenzymatic role of LSD1 in AML. *Nature Chemical Biology*.

[77] Maiques-Diaz A., Spencer G. J., Lynch J. T. (2018). Enhancer activation by pharmacologic displacement of LSD1 from GFI1 induces differentiation in acute myeloid leukemia. *Cell Reports*.

[78] Maes T., Mascaró C., Tirapu I. (2018). ORY-1001, a potent and selective covalent KDM1A inhibitor, for the treatment of acute leukemia. *Cancer Cell*.

[79] Yatim A., Benne C., Sobhian B. (2012). NOTCH1 nuclear interactome reveals key regulators of its transcriptional activity and oncogenic function. *Molecular Cell*.

[80] Lobry C., Oh P., Aifantis I. (2011). Oncogenic and tumor suppressor functions of Notch in cancer: it’s NOTCH what you think. *Journal of Experimental Medicine*.

[81] Li Y., Deng C., Hu X. (2012). Dynamic interaction between TAL1 oncoprotein and LSD1 regulates TAL1 function in hematopoiesis and leukemogenesis. *Oncogene*.

[82] Su S.-T., Ying H.-Y., Chiu Y.-K., Lin F.-R., Chen M.-Y., Lin K.-I. (2009). Involvement of histone demethylase LSD1 in Blimp-1-mediated gene repression during plasma cell differentiation. *Molecular and Cellular Biology*.

[83] Mould D. P., McGonagle A. E., Wiseman D. H., Williams E. L., Jordan A. M. (2015). Reversible inhibitors of LSD1 as therapeutic agents in acute myeloid leukemia: clinical significance and progress to date. *Medicinal Research Reviews*.

[84] Højfeldt J. W., Agger K., Helin K. (2013). Histone lysine demethylases as targets for anticancer therapy. *Nature Reviews. Drug Discovery*.

[85] Milletti F., Cheng W.-Y., Maes T. (2016). Abstract 4708: neuroendocrine gene transcript expression is associated with efficacy to lysine-specific demethylase-1 inhibitor RG6016 in small cell lung cancer-derived cell lines. *Cancer Research*.

[86] Mohammad H., Smitheman K., Cusan M. (2013). Inhibition of LSD1 as a therapeutic strategy for the treatment of acute myeloid leukemia. *Blood*.

[87] Lee S. H., Stubbs M., Liu X. M. (2016). Discovery of INCB059872, a novel FAD-directed LSD1 inhibitor that is effective in preclinical models of human and murine AML. *Cancer Research*.

[88] Bauer T. M., Besse B., Martinez-Marti A. (2019). Phase I, open-label, dose-escalation study of the safety, pharmacokinetics, pharmacodynamics, and efficacy of GSK2879552 in relapsed/refractory SCLC. *Journal of Thoracic Oncology*.

[89] ClinicalTrials.gov (2014). A phase I dose escalation study of GSK2879552 in subjects with acute myeloid leukemia (AML). https://clinicaltrials.gov/ct2/show/NCT02177812/2014.

[90] Hollebecque A., de Bono J. S., Salvagni S. (2019). Phase I study of CC-90011 in patients with advanced solid tumours (STs) and relapsed/refractory non-hodgkin lymphoma (R/R NHL). *Annals of Oncology*.

[91] ClinicalTrials.gov (2020). IMG-7289 in patients with essential thrombocythemia. https://clinicaltrials.gov/ct2/show/NCT04254978.

[92] Imago BioSciences (2020). Imago biosciences granted access by European medicines agency to PRIME scheme for IMG-7289 (bomedemstat) in myelofibrosis. https://www.imagobio.com/imago-biosciences-granted-access-by-european-medicines-agency-to-prime-scheme-for-img-7289-bomedemstat-in-myelofibrosis/2020.

[93] Imago BioSciences (2018). Imago biosciences completes enrollment in phase 1/2a study of IMG-7289 in acute myeloid leukemia and myelodysplastic syndrome. https://www.imagobio.com/imago-biosciences-completes-enrollment-in-phase-1-2a-study-of-img-7289-in-acute-myeloid-leukemia-and-myelodysplastic-syndrome/2018.

[94] Wass M., Göllner S., Besenbeck B. (2020). A proof of concept phase I/II pilot trial of LSD1 inhibition by tranylcypromine combined with ATRA in refractory/relapsed AML patients not eligible for intensive therapy. *Leukemia*.

[95] CenterWatch (2018). Study of sensitization of non-M3 AML blasts to ATRA by epigenetic treatment with tranylcypromine (TCP). https://www.centerwatch.com/clinical-trials/listings/167808/acute-myeloid-leukemia-study-sensitization-non-m3-aml/?&radius=50/2018.

[96] BioSpace (2019). ORYZON presents new efficacy data from its phase II trial ALICE investigating iadademstat in AML. https://www.biospace.com/article/releases/oryzon-presents-new-efficacy-data-from-its-phase-ii-trial-alice-investigating-iadademstat-in-aml-/2019.

[97] ORYZON (2018). ORYZON receives approval to start CLEPSIDRA: a phase IIa clinical trial in small cell lung cancer with iadademstat (ORY-1001). https://www.oryzon.com/en/news-events/news/oryzon-receives-approval-start-clepsidra-phase-iia-clinical-trial-small-cell-lung.

